# RU486 Mitigates Hippocampal Pathology Following Status Epilepticus

**DOI:** 10.3389/fneur.2016.00214

**Published:** 2016-11-28

**Authors:** Aynara C. Wulsin, James P. Herman, Steve C. Danzer

**Affiliations:** ^1^Department of Psychiatry and Behavioral Neuroscience, College of Medicine, University of Cincinnati, Cincinnati, OH, USA; ^2^Neuroscience Graduate Program, College of Medicine, University of Cincinnati, Cincinnati, OH, USA; ^3^Department of Anesthesia and Pediatrics, Cincinnati Children’s Hospital Medical Center, Cincinnati, OH, USA

**Keywords:** RU486, mifepristone, status epilepticus, hippocampus, mossy cells

## Abstract

Status epilepticus (SE) induces rapid hyper-activation of the hypothalamo–pituitary–adrenocortical (HPA) axis. HPA axis hyperactivity results in excess exposure to high levels of circulating glucocorticoids, which are associated with neurotoxicity and depression-like behavior. These observations have led to the hypothesis that HPA axis dysfunction may exacerbate SE-induced brain injury. To test this hypothesis, we used the mouse pilocarpine model of epilepsy to determine whether use of the glucocorticoid receptor antagonist RU486 can attenuate hippocampal pathology following SE. Excess glucocorticoid secretion was evident 1 day after SE in the mice, preceding the development of spontaneous seizures (which can take weeks to develop). RU486 treatment blocked the SE-associated elevation of glucocorticoid levels in pilocarpine-treated mice. RU486 treatment also mitigated the development of hippocampal pathologies induced by SE, reducing loss of hilar mossy cells and limiting pathological cell proliferation in the dentate hilus. Mossy cell loss and accumulation of ectopic hilar cells are positively correlated with epilepsy severity, suggesting that early treatment with glucocorticoid antagonists could have anti-epileptogenic effects.

## Introduction

Temporal lobe epilepsy (TLE) is commonly modeled by chemically inducing status epilepticus (SE) in rodents. SE induces widespread brain damage and neuronal restructuring, which lead to the onset of spontaneous seizures (epilepsy) a few weeks later. In particular, the loss of glutamatergic hilar mossy cells and the misplacement of newly generated dentate granule cells (DGCs) to the hilus (HIL) constitute key hippocampal pathologies that occur in synchrony with – or even precede – the development of epilepsy (1–5). Mossy cells mediate feedback inhibition of hippocampal DGCs by activating GABAergic basket cells (6), although direct connections between excitatory mossy cells and granule cells make their net contribution to dentate excitability complex (2). Ectopic granule cells, on the other hand, are hypothesized to destabilize the hippocampal network, promoting hyperexcitability (2, 7). Notably, mossy cell loss and ectopic migration of granule cells positively correlate with epilepsy severity (8), and ablating ectopic cells reduces seizure frequency (9, 10).

The hypothalamo–pituitary–adrenocortical (HPA) axis is rapidly activated by SE, manifested as glucocorticoid basal hypersecretion (11). Animal studies demonstrate that exposure to excess glucocorticoids can be detrimental in the context of epilepsy, leading to increased brain excitability (12–14) and potential damage (15). Conversely, the glucocorticoid synthesis inhibitor metyrapone reduces neuronal injury when given simultaneously with the SE-inducing convulsant kainic acid (16).

Status epilepticus increases glucocorticoid levels, and glucocorticoids can damage the brain. Collectively, these data suggest that blockade of glucocorticoid signaling may be beneficial in preventing hippocampal damage following seizures. Here, we determined whether treatment with the glucocorticoid receptor antagonist RU486, beginning 4 h after the onset of SE, can mitigate pathological changes associated with TLE.

## Materials and Methods

### Animals

All animal procedures were approved by the Institutional Animal Care and Use Committee of the Children’s Hospital Research Foundation and conform to NIH guidelines. Forty-eight 2-month-old C57Bl/6 male mice were obtained from Charles River. All mice received a subcutaneous (s.c.) injection of methyl scopolamine nitrate (1 mg/kg) followed by 420 mg/kg pilocarpine (*n* = 32) or saline (*n* = 16) 15 min later (8). Three hours after the onset of SE, mice received two doses of diazepam (10 mg/kg) at 15-min intervals to diminish seizure activity. Control animals also received diazepam. Pretreatment body weight was maintained using sterile Ringer’s solution s.c. to avoid potential SE-related dehydration. Mice treated with pilocarpine were monitored for behavioral seizures and the onset of SE. Only mice that experienced two or more convulsive seizures and exhibited constant behavioral seizure activity (myoclonic jerks, immobility, head bobbing, etc.) for 3 h were considered to be in SE and used in the study.

Following SE, mice were housed in a 32°C incubator overnight. The next morning, mice were returned to their home cages with *ad libidum* food and water under a 14/10-h light/dark cycle. The final number of SE mice (*n* = 14) reflects 44% survival of pilocarpine-treated mice. No mortality occurred in the saline-treated group. One hour following the last administration of diazepam, mice were randomly assigned to treatment with either RU486 (20 mg/kg s.c., Sigma-Aldrich, MO, USA) or vehicle (propylene glycol). Treatment was given every morning for seven consecutive days. Groups were generated as follows: (1) SE + vehicle (*n* = 6), (2) SE + RU486 (*n* = 8), (3) no-SE + vehicle (*n* = 8), and (4) no-SE + RU486 (*n* = 8). The RU486 dose was selected based on prior studies (17). Baseline corticosterone levels were measured *via* radioimmunoassay (MP Biomedicals, Orangeburg, NY, USA) using blood collected by tail nick between 9:00 and 10:00 a.m. on days 1, 4, and 7 after SE. Beginning 4 days after pilocarpine or saline treatment, all mice received the first of three daily injections of 5-bromo-2′-deoxyuridine (BrdU; 50 mg/kg s.c., Sigma-Aldrich) to label dividing cells (18). Mice were perfused with 2.5% paraformaldehyde on the eighth day, and brains were prepared for histological analysis (18).

### RU486

RU486 is a synthetic steroid (17 beta-hydroxy-11 beta-[4-dimethylamino phenyl] 17 alpha-[1-propynyl]estra-4,9-dien-3-one) capable of antagonizing the glucocorticoid (doses 5 mg/kg in rodents) (19) and progesterone (doses 1.5 mg/kg in rodents) (20) receptors *in vitro* and *in vivo*. It also has moderate androgen receptor antagonist properties *in vitro* (21); however, *in vivo* effects have only been noted in the testes of rats and not mice at repeated doses >25 mg/kg (22). RU486 is active as an anti-glucocorticoid at doses >5 mg/kg and readily crosses the blood–brain barrier at doses >10 mg/kg (23). RU486 binds GR *in vivo* 3.7× more potently than dexamethasone (24). RU486 is metabolized within 1–2 h following administration (half-life ~1 h) (25). The rate of clearance was calculated as 1.5–6 L/h/kg in rats (data not available for mice) (22). Autoradiography studies in male and female rats demonstrate selective targeting of brain regions including the hippocampus (25). For this study, a 20-mg/kg s.c. dose was selected based on prior work in mice, showing tolerability (22) and a dampening of glucocorticoid-induced effects in the hippocampus (17).

### Immunohistochemistry

Free-floating brain sections (35 μm) were used for immunohistochemistry. For BrdU immunostaining, tissue was placed in 50% formamide and 1× sodium citrate buffer (0.3M NaCl, 0.03M sodium citrate) at 67°C for 2 h, followed by 30-min treatment with 2 N HCl at 37°C. Subsequently, tissue was incubated for 1 h in blocking solution (0.1% BSA, 4% NGS, 0.2% triton-X) followed by overnight incubation in rat anti-BrdU (1:800 AbD Serotec Cat# MCA2060T, RRID:AB_10015293) at 4°C. For GluR2/3 immunostaining, tissue was incubated in the same blocking solution for 1 h, followed by overnight incubation in rabbit anti-GluR2/3 (1:500 Millipore Cat# 07-598, RRID:AB_11213931). Biotinylated anti-rat (1:500 Vector Laboratories Cat# BA-9400, RRID:AB_2336202) and anti-rabbit (1:500 Vector Laboratories Cat# BA-1100, RRID:AB_2336201) secondary antibodies were used for BrdU and GluR2/3 immunostaining, respectively. This step was followed by incubation in ABC reagent (1:800, Vector Laboratories Cat# PK-7100, RRID:AB_2336827) and diaminobenzidine (Sigma-Aldrich) for peroxidase detection (26). Sections were mounted on gelatin-coated slides, co-stained with cresyl violet, dehydrated, and coverslipped.

### Cell Counts

Quantification of BrdU+ and GluR2/3+ cells was done using a modification of the optical fractionator method as previously described (27). Briefly, a 1-mm thick region of dorsal hippocampus (bregma −2.80 to −3.80) was sampled by examining every sixth section (35 μm/section; four sections/mouse). Two regions of interest were examined: the dentate granule cell body layer and the HIL (Figure 2A). The dentate granule cell body layer included the subgranular zone, defined as an approximately two cell thick region between the HIL and the cell body layer. The HIL encompassed the area created between the upper and lower blades of the dentate, but excluding the subgranular zone and the portion of the CA3 pyramidal cell layer that protrudes into the HIL (28). Stereoinvestigator 5.05 (MicroBrightfield, Williston, VT, USA) set up on an Olympus BX-60 microscope equipped with a CCD video camera (HV-C20 Hitachi, San Jose, CA, USA) was used for imaging and quantification. A 5× objective was used to trace all contours, and a 60× NA 1.35 objective was used to quantify all immunopositive cells located within the region of interest. Measured tissue thickness was ~15 μm (~50% shrinkage of 35-μm sections). Optical dissector height was set at 11 μm with a 2-μm guard zone. Mossy cells were distinguished from other glutamatergic hilar neurons by their larger size (>30 μm diameter) (2). Estimated volumes were obtained using Cavalleri’s principle [sum of section areas × (15 μm × 6)] (29). Total estimated cell counts per mouse were obtained by adding the number of counted cells in each section and multiplying by six. Final values are reported as density (cells per cubic millimeter).

### Statistical Methods

Mice were randomized to treatment groups to prevent selection bias. All immunohistochemical data were analyzed by two-way ANOVA, with condition (pilocarpine vs. saline) and treatment (RU486 vs. vehicle) as factors. Three-way repeated measures ANOVA was used to analyze baseline levels of plasma corticosterone with time (days 1, 4, and 7), treatment, and SE exposure as factors. Expected interactions were tested by Student Newman–Keuls *post hoc* analysis. Statistical significance was set at *p* ≤ 0.05. Data that violated normality assumptions were normalized by square-root transformation. Statistica software was used to perform three-way repeated measures ANOVA, while SigmaPlot (Version 13.0, Systat Software Inc.) was used for other analyses.

## Results

### RU486 Treatment Reduces Corticosterone Levels Following SE

**Figure 1:**
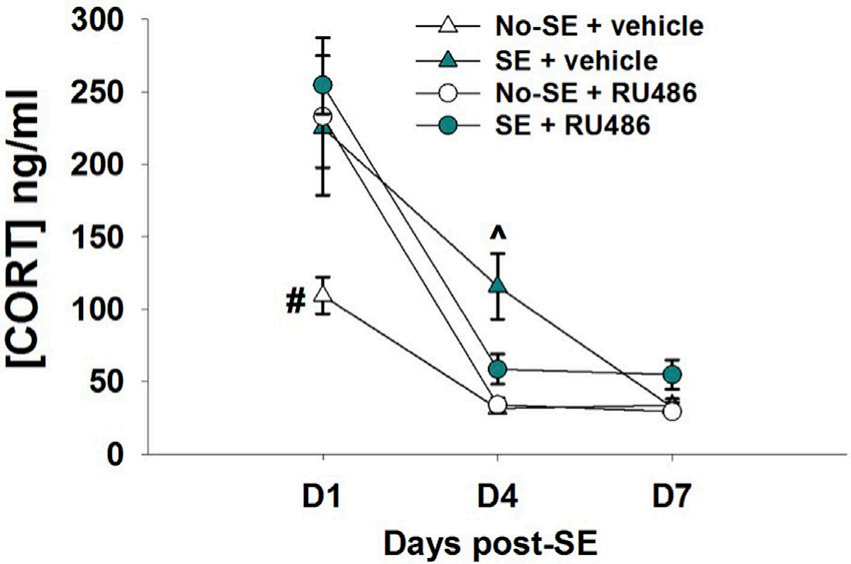
**RU486 decreases morning baseline corticosterone in post-SE mice**. Mice show increased corticosterone morning baseline secretion 24 h post-SE relative to no-SE vehicle-treated mice (^#^*p* < 0.05, no-SE vehicle different from all other groups). RU486 treatment reduced baseline corticosterone secretion in SE mice when compared to SE vehicle-treated mice (^^^*p* < 0.05, SE vehicle different from all other groups). Data presented as mean ± SEM, *n* = 6–8 mice per group.

Corticosterone levels were increased 1 and 4 days after pilocarpine-induced SE relative to no-SE + vehicle mice. RU486 treatment was ineffective at reducing corticosterone levels at the 1-day time point, but levels in SE + RU486 mice were similar to non-SE controls (significantly lower than SE + vehicle mice) 4 days after SE {experimental condition × treatment × time interaction [*F*(2,52) = 5.8019, *p* = 0.0053], *post hoc p* < 0.001} (Figure 1). In addition, RU486 treatment alone increased morning circulating corticosterone levels relative to vehicle-treated mice (*post hoc p* < 0.001) at day 1. This phenomenon has been previously observed in human and rodent studies and is thought to be due to the initial blockade of GR, resulting in greater amounts of circulating corticosterone secondary to an initial disruption of glucocorticoid-mediated negative feedback. At the 7-day time point, morning baseline corticosterone levels were statistically identical in all four groups.

### RU486 Treatment Mitigates Hilar Cell Proliferation

**Figure 2:**
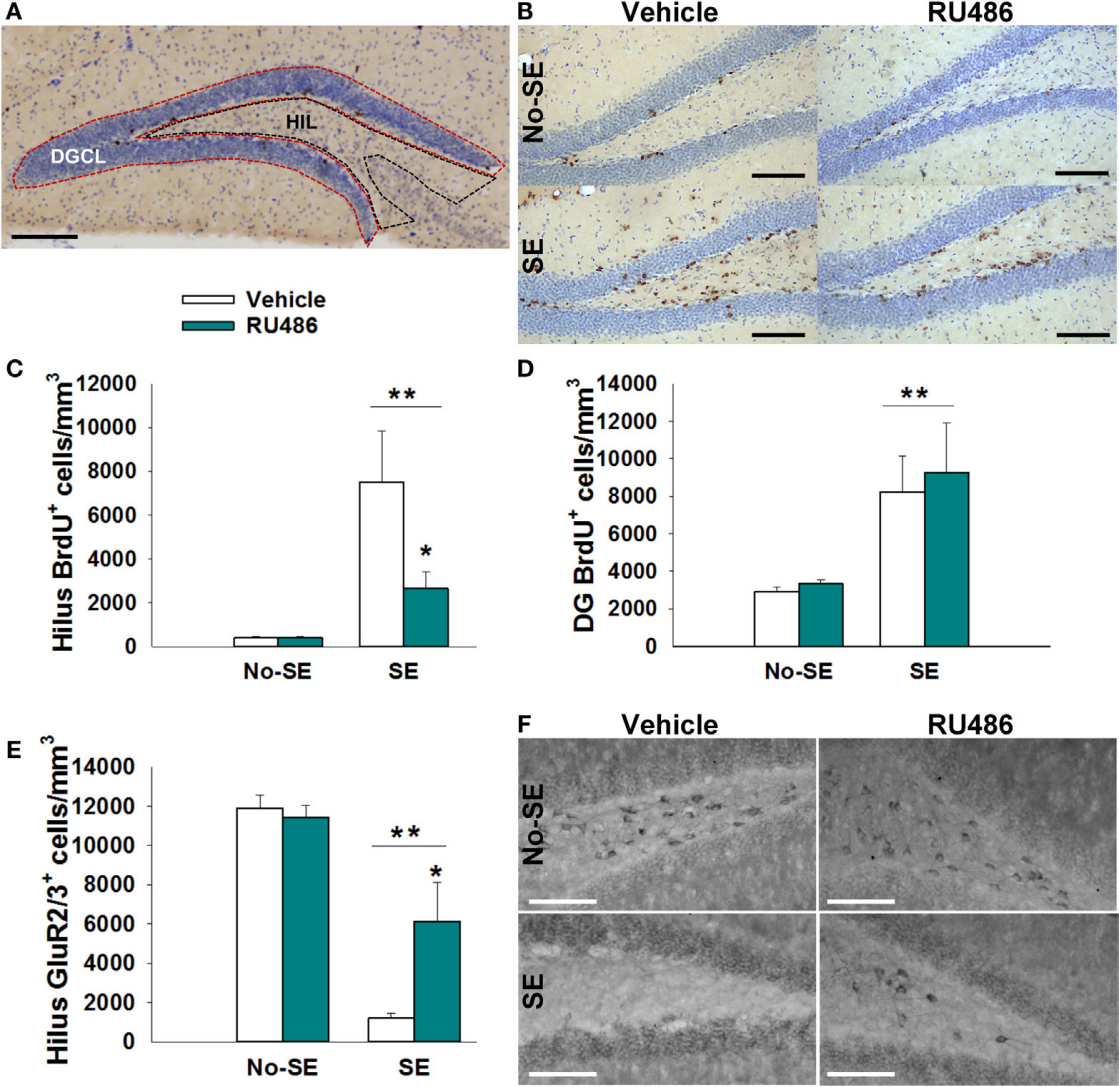
**RU486 reduced mossy cell loss and hilar BrdU+ neurons in the hippocampus of post-SE mice**. **(A)** Micrograph depicting the regions analyzed: dentate granule cell body layer (DGC) and the hilus (HIL). Scale bar = 200 μm. **(B)** Micrograph showing examples of BrdU+ staining. Scale bars = 100 μm. **(C)** SE leads to an increased number of BrdU+ cells in the dentate hilus (**p < 0.01 main effect of SE). RU486 treatment reduced the number of BrdU+ cells in the dentate hilus of post-SE mice relative to vehicle-treated post-SE mice (*p < 0.05, RU486 different from vehicle within SE). **(D)** SE leads to an increase in BrdU+ cells in the dentate gyrus relative to control mice (**p < 0.01 main effect of SE). **(E)** SE leads to loss of GluR2+ cells in the dentate hilus relative to control mice (**p < 0.01, main effect of SE). RU486 treatment preserves greater numbers of GluR2+ cells in the dentate hilus of post-SE mice relative to their vehicle-treated counterparts (*p < 0.05, RU486 different from vehicle within SE). **(F)** Micrograph depicting GluR2 immunohistochemistry in the dentate hilus. Scale bars = 100 μm. All data presented as mean ± SEM, *n* = 6–8 mice.

Status epilepticus increased the density of BrdU+ cells in the dentate HIL by 17-fold in SE + vehicle mice relative to both non-SE groups (*post hoc p* < 0.05). RU486 treatment reduced this increase by 35% {experimental condition × treatment interaction [*F*(1,23) = 8.619, *p* = 0.007], *post hoc p* < 0.05}. Although the SE + RU486 group still had significantly more BrdU+ cells/HIL than the non-SE groups, counts were significantly reduced relative to SE + vehicle mice (*post hoc p* < 0.05) (Figures 2B,C). RU486 treatment alone (no-SE + RU486) had no effect on the density of BrdU+ hilar cells relative to the no-SE + vehicle group. Finally, SE also increased the density of BrdU+ cells within the dentate granule cell layer and subgranular zone compared to both groups of control mice. RU486 treatment had no effect on increased cell proliferation in these regions {main effect of experimental condition [*F*(1,23) = 24.627, *p* < 0.001]} (Figure 2D).

### RU486 Treatment Mitigates SE-Induced Mossy Cell Loss

Status epilepticus produced an 88% reduction in the density of GluR2/3+ mossy cells in the HIL relative to control mice {main effect of pathology [*F*(1,21) = 70.448, *p* < 0.001]} (Figures 2E,F). In SE mice treated with RU486, only a 48% reduction in mossy cells/HIL was observed. While this reduction was still significant relative to non-SE groups, RU486 treatment clearly mitigated mossy cell loss relative to SE + vehicle-treated mice {pathology × treatment interaction [*F*(1,21) = 10.453, *p* = 0.004], *post hoc p* < 0.05}. RU486 treatment alone (no-SE + vehicle) had no effect on mossy cell numbers.

## Discussion

The present study demonstrates that treatment with GR antagonist RU486 effectively blocks the SE-associated elevation of glucocorticoid levels in pilocarpine-treated mice, reduces hilar mossy cell loss, and mitigates cell proliferation in the HIL. Taken together, these findings indicate that GR blockade holds promise as a novel treatment for SE-induced brain injury.

The present findings extend prior work showing pronounced dysregulation of corticosterone levels following SE. Increased morning corticosterone secretion 24 h after SE has been observed previously (11), consistent with the current study. Here, we further demonstrate that SE-induced hypersecretion of corticosterone persists for at least 4 days but returns to baseline by 7 days. These results reveal a transient initial phase of hypersecretion. Interestingly, elevated corticosterone secretion has also been observed in rats 6 weeks after SE (30). These findings imply bimodal changes in corticosterone levels following SE, with early and late periods of hypersecretion.

Treatment with RU486 blocked the increase in corticosterone levels evident in untreated animals 4 days after SE. The mechanism by which chronic RU486 treatment normalizes corticosterone levels is unknown. It has been hypothesized, however, that prolonged antagonism of GR may result in upregulation of the mineralocorticoid receptor in critical hypothalamic and limbic structures. Because RU486 has no affinity for the mineralocorticoid receptor (31), glucocorticoid binding to mineralocorticoid receptors in these stress-regulatory regions may induce resetting of the HPA axis and reinstate control of baseline secretion (32–34). In addition, increased glucocorticoid binding to the mineralocorticoid receptor may oppose some of the toxic effects of GR binding (35, 36) and SE, thereby preserving key HPA axis feedback pathways.

In addition to actions on GR, RU486 also acts as a progesterone receptor antagonist (31). Although we cannot exclude the possibility that progesterone receptor antagonism is important, we favor the interpretation that reduced hippocampal pathology reflects GR antagonisms, as progesterone receptors are not widely expressed in the mouse hippocampus (37). Nonetheless, additional studies with more selective approaches will be needed to unambiguously rule out off-target effects.

RU486 treatment reduced mossy cell loss in the dentate HIL, suggesting that GR signaling during the hours and days following the insult may contribute to the development of hippocampal pathology. Findings are reminiscent of prior work showing that RU486 protects against CA1 neuronal loss following traumatic brain injury (35). GR is expressed in all hippocampal subfields and in mossy cells (38–40). Glucocorticoids, therefore, may act directly on vulnerable neurons to influence their survival. Alternatively, GR blockade on DGCs may indirectly reduce mossy cell loss. Glucocorticoids promote hypersecretion of glutamate by granule cell mossy fiber axons as early as 24 h following pilocarpine-induced SE, which can be blocked by treatment with RU486 (41). Granule cell axons directly innervate mossy cells and may contribute to excitotoxic loss of these neurons.

Status epilepticus results in large increases in cell proliferation, cell survival, and neurogenesis (42, 43). Consistent with these studies, post-SE mice had increased numbers of BrdU+ cells located in the subgranular zone and granule cell layer of the dentate gyrus. RU486 treatment did not have an effect in the number of BrdU+ cells in either of these regions. RU486 did, however, reduce the density of BrdU+ cells located in the dentate HIL. SE can induce astroglial and microglial proliferation in the HIL (44, 45), as well as the accumulation of ectopic, newly generated granule cells (46–48). Reductions in cell proliferation in the HIL imply that RU486 mitigates some or all of these proliferative changes. Indeed, glucocorticoids have been implicated in regulating the migration, maturation, and functional integration of DGCs (49, 50). Similarly, RU486 has been shown to prevent hippocampal astrogliosis in mice (51). Thus, glucocorticoid blockade following SE may prevent the aberrant migration of newborn granule cells into the dentate HIL and/or decrease astrogliosis.

Finally, it is important to note that RU486 administration to control mice did not alter any of the measures examined here and has been previously found to exert little impact on cell proliferation, survival, and inflammation in control subjects (32, 52–54). Our results are in agreement with other studies suggesting that the actions of RU486 may be specific to the pathological, high corticosterone environment (31). This specificity reflects the ability of RU486 to preferentially bind the GR over the mineralocorticoid receptor (31). Since GR’s are only fully occupied when corticosterone levels are elevated (55), the impact of blockade is most pronounced under these conditions.

## Conclusion

The current study demonstrates that in the pilocarpine model of epilepsy, SE results in the rapid elevation of glucocorticoids. Treatment with the glucocorticoid antagonist RU486 following the insult blocks the SE-induced elevation of glucocorticoid baseline secretion, mitigates mossy cell loss, and reduces aberrant cell proliferation in the HIL. Our laboratory and others have implicated mossy cell loss and aberrant neuron proliferation in epileptogenesis, *via* mechanisms that result in hippocampal hyperexcitability (1–5). In addition, astrogliosis and brain inflammation are also associated with the pathophysiology of epilepsy (56). Together, our findings suggest that blockade of glucocorticoid signaling may be beneficial in preventing hippocampal damage following SE. To advance this line of investigation, it will be important in future studies to examine extended survival times so that the impact of RU486 on seizure frequency and animal behavior can be assessed. Time course studies to determine how long treatment can be delayed before it loses efficacy will also be important. Mossy cell loss may occur over days and weeks (57), but since this is a non-regenerative cell population, at some point loss will be irreversible. Finally, although the use of an FDA-approved drug like RU486 has obvious advantages for rapid translation, the use of more specific glucocorticoid receptor antagonists will be important to experimentally isolate glucocorticoid receptor-mediated from progesterone receptor-mediated effects.

## Author Contributions

AW participated in experimental design, animal handling, analysis of neuroendocrine and tissue data as well as all statistical analyses for this study. In addition, AW wrote the manuscript and created all figures. JH and SD assisted with experimental design and provided editorial assistance for the manuscript.

## Conflict of Interest Statement

The authors declare that the research was conducted in the absence of any commercial or financial relationships that could be construed as a potential conflict of interest.
